# Refractory Sweet Syndrome Treated with Anakinra

**DOI:** 10.7759/cureus.4536

**Published:** 2019-04-24

**Authors:** Zainab Shahid, Ricci Kalayanamitra, Ravi Patel, Andrew Groff, Rohit Jain

**Affiliations:** 1 Internal Medicine, Lake Erie College of Osteopathic Medicine, Erie, USA; 2 Emergency Medicine, Penn State Health Milton S. Hershey Medical Center, Hershey, USA; 3 Internal Medicine, Penn State Health Milton S. Hershey Medical Center, Hershey, USA

**Keywords:** sweet syndrome, acute febrile neutrophilic dermatosis, anakinra, chronic, refractory, recalcitrant

## Abstract

Sweet syndrome, otherwise known as acute febrile neutrophilic dermatosis, is an uncommon disorder characterized by the abrupt onset of painful cutaneous lesions, often with fever and leukocytosis, in patients with underlying infection, malignancy, pregnancy, or drug exposure. We present a case of a young female with long-standing Sweet syndrome refractory to standard treatments and several immunomodulators whose symptoms were ultimately controlled with anakinra.

## Introduction

Sweet syndrome was first reported in 1964 in the *British Journal of Dermatology* by Dr. Robert Douglas Sweet, who described eight women with skin eruptions, fever, and neutrophilic leukocytosis [1]. Sweet syndrome has since been associated with a range of disorders and is currently categorized into three subtypes based on etiology, namely as classical Sweet syndrome, malignancy-associated Sweet syndrome, and drug-induced Sweet syndrome. The first-line treatment for this condition is high-dose systemic corticosteroid therapy. Commonly used alternative medications include colchicine, dapsone, and potassium iodide [2]. There is a dearth of literature on cases of Sweet syndrome refractory to standard treatment, and even fewer reports of Sweet syndrome treated with anakinra [2-14].

## Case presentation

A 19-year-old female with a past medical history significant for long-standing Sweet syndrome requiring multiple emergency department visits presented to the emergency department with generalized muscle and joint pain and a diffuse outbreak of papules and vesicles. Her condition had been refractory to corticosteroids, colchicine, dapsone, adalimumab, abatacept, infliximab, etanercept, azathioprine, leflunomide, lenalidomide, and methotrexate, and was being managed with tocilizumab. The patient's condition was consistent with an acute exacerbation of Sweet syndrome and she was treated and discharged on a 40 mg prednisone taper.

Two days after this visit, the patient returned to the emergency department with worsening muscle and joint pain and a new eruption of diffuse skin lesions. Vital signs revealed a temperature of 36.7°C, pulse of 104 beats per minute, blood pressure of 139/90 mmHg, respiratory rate of 18, and oxygen saturation of 99% on room air. Physical examination revealed tender, erythematous, and ulcerating papules and pustules scattered over the trunk, bilateral upper and lower extremities, and face. Laboratory workup was notable for a leukocyte count of 16,800 cells/μL in the setting of recent steroid use, platelet count of 411,000 platelets/μL, c-reactive protein of 1.15 mg/L, and erythrocyte sedimentation rate of 37 mm/h.

The patient was admitted and started on 250 mg of methylprednisolone twice per day and colchicine 0.6 mg twice per day but continued to develop new lesions on her face, lower back, and tongue. Pain management was attempted with acetaminophen, gabapentin, tizanidine, duloxetine, and toradol, and ultimately the patient’s pain was controlled with hydromorphone 4 mg taken every three hours as needed. The patient was given 100 mg of anakinra and her skin lesions began to improve. She was discharged with oral pain medications after a seven-day inpatient stay and followed up with outpatient rheumatology for continued treatment.

## Discussion

Sweet syndrome is an uncommon disorder and therefore it is often missed on initial presentation. Specific diagnostic criteria have been established for each subtype of this syndrome (Tables 1-2). Classical Sweet syndrome represents the majority of cases and develops most commonly in individuals between the ages of 30 and 60 in the settings of inflammatory bowel disease, pregnancy, or a few weeks after an upper respiratory or gastrointestinal infection [2]. Malignancy-associated Sweet syndrome can occur before, after, or concurrently with either a solid tumor or hematologic malignancy. This subtype tends to affect older patients, with one study estimating the average age at diagnosis of 68 years old [15]. Drug-induced Sweet syndrome usually develops a few weeks after initial drug exposure and is most commonly due to granulocyte-colony stimulating factor (G-CSF). All subtypes of this syndrome have a female predominance [2].

**Table 1:** Diagnostic criteria for classical and malignancy-associated Sweet syndrome The diagnosis of classical or malignancy-associated Sweet syndrome requires both of the major criteria and two of the four minor criteria to be met [2].

Major criteria	Minor criteria
Abrupt onset of painful erythematous plaques or nodules	Pyrexia >38 C
Histopathologic evidence of a dense neutrophilic infiltrate without evidence of leukocytoclastic vasculitis	Association with underlying hematologic or visceral malignancy, inflammatory disease, or pregnancy OR preceded by an upper respiratory or gastrointestinal infection or vaccination
	Excellent response to treatment with systemic corticosteroids or potassium iodide
	Abnormal laboratory values at presentation (three of four); erythrocyte sedimentation rate >20 mm/h; positive c-reactive protein; >8,000 leukocytes; >70% neutrophils

**Table 2:** Diagnostic criteria for drug-induced Sweet syndrome The diagnosis of drug-induced Sweet syndrome requires all five criteria to be met [2].

Major criteria
Abrupt onset of painful erythematous plaques or nodules
Histopathologic evidence of a dense neutrophilic infiltrate without evidence of leukocytoclastic vasculitis
Pyrexia >38 C
Temporal relationship between drug ingestion and clinical presentation OR temporally-related recurrence after oral challenge
Temporally related resolution of lesions after drug withdrawal or treatment with systemic corticosteroids

The pathogenesis of this disorder is not well understood but is theorized to be at least partially due to cytokine dysregulation. G-CSF is believed to contribute to this disorder because it increases circulating neutrophils. In support of this, one study comparing patients with active and inactive Sweet syndrome revealed elevated production of G-CSF in patients with active Sweet syndrome [16]. In another study, interleukin-1, interleukin-2, and interferon-gamma were found to be elevated in patients with active Sweet syndrome [17]. These findings are supported by the efficacy of certain immunomodulatory drugs in cases of refractory Sweet syndrome and by the fact that interleukin-1 induces endothelial cell release of G-CSF [3-14,18].

Our patient’s case is distinct from previous reports because she failed both standard corticosteroid and colchicine therapies, and failed trials of multiple immunomodulatory medications. Her symptoms were eventually controlled with anakinra, an interleukin-1 receptor antagonist, and this response further supports the likelihood of an interleukin-1 pathway in the pathogenesis of Sweet syndrome. It also opens the possibility that our patient’s case represents an example of an interleukin-1 mediated variant of Sweet syndrome and that multiple variants of this syndrome may exist, especially because the patient’s symptoms were refractory to the interleukin-6 receptor inhibitor tocilizumab. It is important that further research is conducted on Sweet syndrome in a large cohort of patients in order to determine the pathogenesis as well as investigate the possibility of multiple variants.

## Conclusions

Sweet syndrome should be considered in patients presenting with an abrupt onset of a diffuse rash with possible fever and leukocytosis, especially in the settings of recent drug use, malignancy, inflammatory disorder, and recent infection. In addition, anakinra should be considered in patients whose symptoms are unresponsive to both standard treatments and immunomodulators because although the syndrome itself is not fatal, it is very painful and often requires opioid therapy for appropriate pain control.
